# Practical considerations for a library's research data management services: the case of the National Institutes of Health Library

**DOI:** 10.5195/jmla.2021.995

**Published:** 2021-07-01

**Authors:** Soojung Kim, Sue Yeon Syn

**Affiliations:** 1 kimsoojung@jbnu.ac.kr, Department of Library and Information Science, Jeonbuk National University, Jeonju, South Korea; 2 syn@cua.edu, Department of Library and Information Science, The Catholic University of America, Washington, DC

**Keywords:** research data management, case study, library services

## Abstract

**Objective:**

This study investigates research data management (RDM) services using a crosstab framework with the National Institutes of Health (NIH) Library as a case study to provide practical considerations for libraries seeking to improve their RDM services.

**Methods:**

We conducted semistructured interviews with four librarians who provide data services at the NIH Library regarding library user characteristics, RDM services provided, RDM infrastructure, and collaboration experiences. Through the analysis of interview transcripts, we identified and analyzed the NIH Library's RDM services according to Online Computer Library Center (OCLC)'s three categories of RDM services and the six stages of the data lifecycle.

**Results:**

The findings show that the two models' crosstab framework can provide an overview of an institution's current RDM services and identify service gaps. The NIH Library tends to take more responsibility in providing education and expertise services while relying more on information technology departments for curation services. The library provides significant support for data creation, analysis, and sharing stages to meet biomedical researchers' needs, suggesting areas for potential expansion of RDM services in the less supported stages of data description, storage, and preservation. Based on these findings, we recommend three key considerations for libraries: identify gaps in current services, identify services that can be supported via partnerships, and get regular feedback from users.

**Conclusion:**

These findings provide a deeper understanding of RDM support on the basis of RDM service categories and the data lifecycle and promote discussion of issues to be considered for future improvements in RDM services.

## INTRODUCTION

In recent decades, the increasing volume of data-driven research and changing funding agency policies for research data management (RDM) have posed significant challenges for researchers who are not well versed in RDM practices and sharing [1, 2]. In response, many libraries have expanded their services to address researchers' growing demand for RDM support.

Several earlier studies discuss the RDM roles of libraries and library services [3–5]. The demand for RDM services has been strong, particularly in the health sector [6]. Accordingly, health science libraries have developed a wide range of RDM services: instruction and consultation for data management plans (DMPs) [7]; training on data analysis tools and online bioinformatics application guides [8]; qualitative data analysis, data visualization, and data wrangling [9]; training on clinical data management [9, 10]; developing data dictionaries and standardized data request forms [11]; and file management, assigning metadata to data, naming variables, and finding an appropriate data repository [12]. A recurring theme characterizing RDM services is collaboration among stakeholders. Meeting the various needs that arise throughout the data lifecycle cannot be assigned to a single unit within an institution. Collaborations and partnerships among libraries, information technology (IT) departments, and other internal or external units are key to the development and success of RDM services [11–14].

This study investigates RDM services using the National Institutes of Health (NIH) Library as a case study and provides practical considerations for libraries seeking to extend their RDM services. The NIH's RDM services were analyzed based on Online Computer Library Center (OCLC)'s RDM service categories [15] and the data lifecycle. In the RDM process, data pass through a series of stages, from creation to preservation and reuse. Given that “the concept of the research data lifecycle plays a central role in organizing and structuring services” [16], analyzing RDM services at each stage of the data lifecycle to identify areas to introduce new services can be useful. Because NIH is one of the world's leading medical centers, it was selected to examine RDM services relating to a wide range of data management scenarios in biomedical research. The NIH Library serves career-level researchers and related staff [17], but their RDM services apply to many academic libraries whose faculty and students are considered professional and future researchers who manage data. This study's findings can be useful for specialized and research libraries that serve researchers, particularly in biomedical fields, that aim to develop or improve their RDM support services.

## METHODS

### Setting

The NIH Library serves the NIH Intramural Research Program, the largest biomedical research institution globally, numbering approximately 1,200 principal investigators, 4,000 postdoctoral fellows, and many other employees. The NIH Library also serves other institutions in the US Department of Health and Human Services [18].

The NIH Library created a data services team in 2013 [18]. In addition to a team leader, two other librarians help implement the team's operational and strategic objectives. Data services librarians work closely with the bibliometrics team of two librarians, which provides bibliometrics services and is actively involved in data services training. Another team related to data services is the editing team, which strategizes with researchers to select target journals for their manuscripts and assists them with creating and formatting datasets for inclusion in publications.

The library has a matrix structure that maintains essential library functions and organizational structures, although cross-functional groups perform much of the work. The data services team, bibliometrics team, editing team, and other units are the library's structural units, but library services or projects can be implemented collaboratively by librarians in multiple units, which allows for flexibility in utilizing librarians' knowledge and enables library resources to be used most effectively.

### Conceptual framework

To develop a systematic approach to characterizing the types of RDM services available at NIH, we borrow from two different models: OCLC's RDM services categories and the data lifecycle model. The OCLC's RDM service categories were identified by characterizing RDM service components in a review of more than a dozen research libraries in North America, Europe, and Australia, with the goal of “provid[ing] a useful heuristic for visualizing the scope of the RDM service space” [15]. The categories include (1) education services that aim to raise awareness of RDM's importance, instruct researchers in basic RDM skills, and introduce RDM tools and resources; (2) expertise services that offer decision support and customized solutions for RDM problems encountered by researchers; and (3) curation services that provide the technical infrastructure and related services that support RDM throughout the research process.

**Figure 1:**
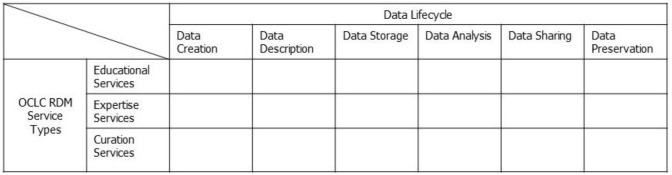
Conceptual framework of the study

Additionally, we used the data lifecycle stages to determine which specific RDM services provide support for particular stages. From four data lifecycle models (DataONE Life Cycle [19], UK Data Service Research Data Lifecycle [20], Digital Curation Centre Curation Lifecycle Model [21], and United States Geological Survey Data Lifecycle [22]), we identified six stages: (1) creation, (2) description, (3) storage, (4) analysis, (5) sharing, and (6) preservation. In the creation stage, researchers generate original data or collect preexisting data from other sources. In the description stage, researchers create appropriate metadata or describe data to support storage and retrieval with the self-generated or collected data in hand. In the storage stage, data are stored appropriately in certain formats. In the analysis stage, data are analyzed to produce results. In the sharing stage, researchers publish data through journals, place them in institutional or public repositories to share with other researchers, or both. In the preservation stage, data are managed for long-term preservation. These six stages of the data lifecycle, together with OCLC's RDM services categories, provide a useful framework for identifying the current coverage and gaps in RDM services provided by the NIH. Figure 1 illustrates the conceptual framework used in this study for RDM services assessment.

### Data collection and analysis

We collected data through semistructured interviews with four librarians, two data services librarians, and two bibliometrics librarians in November 2018 following Institutional Review Board approval from the Catholic University of America. Interview questions asked about library user characteristics, the existing infrastructure of RDM at the library and NIH, the types of RDM services provided at each stage of the data lifecycle, difficulties experienced in providing RDM services, and collaborative experiences (Appendix 1). Interviews were recorded and transcribed for analysis, and the transcripts' coding was aligned with the categories of the interview questions. Predetermined categories derived from OCLC's RDM service categories and the data lifecycle guided the coding process in identifying the types of RDM service offered by the NIH. Under the interview question categories, detailed codes were added to the code categories as they emerged. The two authors coded each transcript individually, compared coding results, and discussed them to resolve discrepancies. The final version of the codes used in the analysis is available in Appendix 2.

Because users' feedback was brought up multiple times during the interviews, the librarians gave us the aggregated results of feedback surveys collected in 2018. We used these data to better explain and interpret the interview content. Surveys were distributed to 372 trainees in selected training classes in 2018 and received 246 responses concerning participants' satisfaction level, expectations for the training classes, and interests in future training sessions.

## RESULTS

### Institutional environment

The NIH Library serves multiple campuses. Also, because NIH comprises twenty-seven separate institutes and centers [23], each of which has its own IT infrastructure, these institutes and centers also handle curation services (data management and servers). This leads to some educational services not being able to be provided to all members of the NIH, although the library's primary RDM services include training classes (see Educational Services section). The NIH's IT department, the Center for Information Technology (CIT), provides integrated services for backup and preservation (see Curation Services section).

The NIH Strategic Plan for Data Science manifests another aspect of the institutional environment that shapes the NIH Library's services. In 2018, the NIH released its first Strategic Plan for Data Science to reinforce the mission of “storing, managing, standardizing and publishing the vast amounts of data produced by biomedical research” [24]. The NIH Library aligns itself closely with the plan, as shown in the data services team leader's remark, “Our goal now is to support the NIH strategic plan.” Because “one of the major pillars of the new Strategic Plan for Data Science is FAIR (Findable, Accessible, Interoperable, and Reusable) Principles” and the data generated in NIH have high potential to be shared with the public, it is essential to teach NIH researchers how to make data usable by others. To do this, librarians promote the use of common data elements (CDEs), which are elements that are common to multiple datasets across different clinical studies [25]. By specifying standardized vocabularies, definitions, and value sets for data, CDEs facilitate the comparison and combination of data from different studies. The NIH provides an extensive repository of CDE collections [26], which librarians encourage researchers to use.

### User characteristics and needs

According to training participants' feedback survey, 39% (N=25 of 64 respondents from June to December) were fellows and trainees working with actual data. Other participants included intramural researchers (17%, N=15), scientific administrators/analysts (11%, N=11), and clinical staff (4.7%, N=3), among others. A possible explanation for the high participation of fellows is that they may not remain at the institute for extended periods, so continuous training may be necessary for newly arriving fellows. Librarians also observed that fellows are more proactive in acquiring new skills that “they can apply during their future careers.”

One interesting and unusual aspect of the NIH Library is that researchers are not required to apply for research funding. Because the library serves only intramural researchers who do not need DMP support, the library does not offer training on writing DMPs, even though this is an essential component of RDM services in many libraries [27]. As one librarian put it, “Since we don't do data management plans, here we look for opportunities where we can talk about best practices … In all of our classes we talk about this best practice for scientific computing, and it's related to storage, data curation, managing data.”

### Collaboration experiences

At the NIH Library, collaboration operates at multiple levels and may involve other library units, other NIH units, or external partners. The NIH Library's matrix structure brings together librarians with diverse skillsets and expertise to work on specific projects. Thus, when the data services team leader announces a new project and puts out a call for support, other librarians may volunteer to join the project team on a provisional basis. Furthermore, librarians “work collaboratively with everybody who does data science at NIH to partner on training.” This interorganizational collaboration enables more efficient use of resources and expertise. For example, the library provides basic statistics training (e.g., Overview of Common Statistical Tests) but has thus far not supported advanced statistics techniques. Since the creation of a chief statistician position in the biostatistics division at the NIH Clinical Center, this person has been able to teach advanced statistics classes for the library.

What is vital in such collaboration is communication. The NIH formed the Training Collaborative Forum, which consists of representatives of various training units within the NIH who administer mandatory training (e.g., Health Insurance Portability and Accountability Act), technical training (e.g., Excel), and for-credit training (e.g., Introduction to Microbiology) [28]. The NIH Library joined this forum to understand the broader training landscape at NIH and avoid potential conflicts in RDM services [28]. Besides coordinating courses and sharing information, the library and other training units collaborated in marketing efforts. An upcoming outreach initiative is that the library and other training units “will have a single orientation presentation for researchers who were not aware of how much support was available to them and whom to contact to get those support services.” The idea is that “only one representative from this joint effort will be going into the labs and that can rotate from any of the divisions which can speak competently about what we do.” Librarians expected to streamline the marketing process and make it more efficient.

External partnerships such as the Carpentries are also particularly beneficial for education services. The Carpentries is a fiscally sponsored project that teaches foundational coding and data science skills to researchers [29] by organizing hands-on workshops that cover concepts and tools researchers can apply to their research. The library brought this training to the NIH by hosting some Carpentries workshops (e.g., Data Carpentry's Genomics Workshop). A librarian commented on the benefit of the workshop with, “What I want to do is a pre-training assessment. So, where people were before they took the training and where they are afterward. We just don't have the manpower, capabilities.” Another mentioned collaboration with software vendors, “We partner with software that we subscribe to, MATLAB, SPSS, JUMP, STATA. We just did a couple of sessions on artificial intelligence and machine learning and data science. We don't have a capacity to do that, so we reached out to our vendors to come in and do training for that.”

### Education services

The educational goal of the Data Services team is “increasing the capacity for everyone at NIH to do data science at some level.” In 2014, the library conducted a survey to assess researchers' perceived data literacy skills and identify topic priorities for training [17]. The results of this survey helped guide the development of classes [28]. Librarians also collect trainee feedback twice a year in June and December to improve their training and plan for the next year's education. Table 1 shows a list of data service–related classes offered during 2018 to 2020. The range of topics is broad, from data analysis to machine learning, with R classes offered most often. In each month of 2018, four R classes were held, resulting in more than 800 people receiving R training that year. Librarians planned to expand R training by adding two more classes in 2019 in response to the feedback survey, in which respondents most frequently recommended R for a new class suggestion.

**Table 1:** Data services classes offered during 2018–2020*

Year	Class titles
2018	Introduction to R Introduction to R and RStudio Introduction to R Data Types APIs in R R Custom Functions R Txt Mning Tidy Data and the R Tidyverse Wrangling Data Using the R Tidyverse Data Visualization Data Visualization in Base R Data Visualization in ggplot MATLAB for Scientists: Working with Images & Video Deep Learning in MATLAB Bibliometrics for portfolio analysis Software Carpentry (Two-Day Workshop) From RePORTER to Web of Science and Incites Overview of Common Statistical Tests Electronic Lab Notebook (ELN) Discussion Top 10 FAIR Data Things Global Sprint
2019†	Introduction to Categorical Data Introduction to MATLAB Hands-on Workshop Openness and Reproducibility Workshop Data Carpentry Genomics Two-Day Workshop
2020†	Meta-Analysis: Quantifying a Systematic Review Statistical Considerations in Preparing Your Manuscript Hands-On Virtual Lab: Machine Learning Introduction to Taxonomies Data Management and Sharing (Planned on September)

* Source: NIH Training & Events. https://www.nihlibrary.nih.gov/training/calendar.

† Classes not available at the time of the interview, but added in later years.

A problem that inevitably arises in the multicampus environment is that offline training sessions are offered only at a limited number of campus locations, where software subscription and access may be restricted. As a tentative solution, the library currently relies on a corporate model of software training for some of its educational services; this entails a “train-the-trainer” model to reach out to instructors and researchers who can attend in-person sessions and later deliver RDM training to their colleagues at other campus locations. Librarians also deliver training in a webinar format for researchers who cannot make it to in-person training sessions. However, they believe that “programming works better […] when you're in an in-person environment,” emphasizing the importance of in-person communication. The training participants' feedback survey showed that 67% of participants in 2018 attended in-person sessions (N=165 of 246), whereas 33% joined the webinars (N=81 of 246).

Education services primarily oriented toward researchers working at the analysis stage mostly involve data analysis applications (e.g., MATLAB). This aligns with researchers' keen interest in learning-specific tools rather than more general best practices in RDM, as evidenced in this remark by one librarian: “Most people come in, and they are like, ‘I want to develop a histogram in ggplot. I don't want to know about best practices for scientific computing. I don't want to know about Introduction to Visualization. I just want to use the tool.'” This remark shows the task-oriented nature of researchers' goals in taking classes and explains why they are application specific.

While their training mainly addresses data analysis needs, librarians have recently begun to emphasize data sharing. In an “Openness and Reproducibility Workshop: A Day of Open Science” in 2019, participants first learned about general issues in data sharing and then how to use the Open Science Framework (OSF), an open-source Web platform that helps researchers create, store, collaborate, and share projects. The workshop focused on OSF's advanced features that facilitate the reproducibility of research data.

### Expertise services

Librarians receive one-on-one consultation requests from researchers on an ad hoc basis, often when researchers visit the Technology Hub at the library, which is collaborative workspace with technology. Researchers request in-person consultations when trying to learn about specific hardware and software or experience difficulties with secondary datasets for their projects. The most common requests for one-on-one consultation are data wrangling to transform raw data from one data format to another. One librarian gave an example of such consultation requests: “A researcher [who] was dealing with six files, and each one was about 3 gigabytes so he couldn't import them into STATA or SPSS; so [librarian's name] and I worked together to sort of split those files up in R, and then do the type of analysis for them and then join them back in a comma-separated file for him to import.” The librarian referred to data wrangling as “the greatest challenge […] Taking data that is not formatted in the way they need for their analysis is the most common request that we get.”

Other consultation requests include asking for data visualization and writing assistance. For data visualization, researcher requests tend to be technical, such as creating particular visualization formats in response to a journal's requirements or for a presentation. The Editing team provides classes that cover topics from generating hypotheses to data presentation and even journal selection. Librarians help researchers with formatting datasets to share with publications. Data formatting requests may also include assistance with adding metadata to datasets.

### Curation services

The NIH's CIT is closely involved in RDM support in terms of the IT infrastructure at the NIH institutional level. It provides “the NIH community with a secure and reliable IT infrastructure and a variety of IT services to support mission-critical research and administration” [30]. Per its Strategic Plan for Data Science, the NIH maximizes all of the resources available, including people and tools. This maximization of resources encourages collaboration among the various NIH units in serving the community, including the CIT and NIH Library.

One of the major services provided by the CIT is a daily, automatic backup service for the servers connected to campus computers. It also manages NIH researchers' institutional servers for data storage and preservation, such as a centralized database called BTRIS (Biomedical Translational Research Information System), where clinical data are stored. Researchers tend to trust such services and infrastructure and rely on them for their data creation, storage, sharing, and preservation. For example, in one lab, a high volume of magnetic resonance imaging (MRI) data are stored on a shared drive on the server so that the lab members granted access to the data could download them with ease. Because MRI data are usually extensive, a shared drive was deemed a more viable option than email or USB, not only for storage but also for transferring data among research collaborators. Moreover, researchers tend not to preserve datasets themselves, but instead rely entirely on CIT for long-term storage. For example, they would contact CIT to retrieve old files instead of digging up their back-up files in an emergency. In addition to CIT, IT departments at each center level also provide curation services.

For the NIH Library, instead of providing technology infrastructure, librarians introduce researchers to alternative options for preserving data, such as Amazon S3, Figshare, or other scientific frameworks, helping them make the right decisions to meet their needs. However, “[Librarians] are not as open with cloud computing being in the public sector as it might be in academia,” probably because of concerns about data security. They leave it to researchers to choose the option that works best for them for preservation. The library's participation in curation services is limited to providing the technology hub and introduction of public repositories, while CIT supports more essential services for data storage and preservation.

### Data lifecycle

We laid out the NIH's RDM services based on OCLC's RDM service categories and the data lifecycle (Table 2).

**Table 2:** NIH's research data management services

	Data creation	Description	Storage	Analysis	Sharing	Preservation
**Education services**		Electronic lab note online discussion		Application-specific classes	Data ethics training (e.g., HIPPA)* Common Data Elements	Introduction of public repositories
**Expertise services**	Technology consultation, Secondary data consultation			Data wrangling Visualization	Visualization Writing services	
**Curation services**	Institutional databases (e.g., CRIS, BTRIS)* Data management hardware and software†	Data management hardware and software† Guide to other resources (e.g., Amazon S3, Figshare)	Institutional servers and databases (e.g., CRIS, BTRIS)* Guide to other resources (e.g., Amazon S3, Figshare)	Application support within institution (mostly domain-specific applications)*	Institutional databases (e.g., CRIS, BTRIS)* Technology hub	Institutional servers*

* Served by non-library units.

† Served collaboratively by the library and non-library units.

The data creation stage is supported by the library mainly through consultation and technological support with databases, hardware, and software. Supporting the data analysis and sharing stages are training sessions, oneon-one consultations, and technological support for hardware, software, and institutional databases. Among the six stages of the data lifecycle, these three seem well provided for in terms of concrete RDM support through various types of services. On the other hand, the data lifecycle's remaining stages (description, storage, and preservation stages) are less well supported. Regarding the data preservation stage, librarians introduce a list of public repositories so that researchers can select an appropriate one for their purposes. CIT supports both back-up storage and archival storage for preservation. For the data description stage, although some efforts such as “Electronic Lab Notebooks (ELN) Online Discussion” provide instruction on using an ELN to describe and document research data, further support for this stage could improve RDM practices. Although training classes at the NIH Library mainly assist with the data analysis stage, there are efforts to increase training in data sharing and other stages of the data lifecycle. Among the three categories of RDM services, the library tends to take more responsibility in the education and expertise services categories while relying more heavily on CIT for the curation services category.

## DISCUSSION

We investigated the NIH's RDM services through the lens of OCLC's RDM services categories and the data lifecycle. Our findings suggest several key practical considerations for librarians and other RDM stakeholders in developing data support services for their research communities.

### Identify gaps in current services

Although many other studies adopt a data lifecycle model as a framework for assessing current data services (e.g., [31–33]), we combined the data lifecycle model with OCLC's RDM services categories to overview current data services at an institution. The two models' crosstab format not only lays out the landscape for the types of data services provided and the stages of RDM they serve, but it also identifies gaps.

We found that the NIH Library provides strong support for data creation, analysis, and sharing via various types of RDM services. This support is understandable considering the needs of biomedical researchers and trends in biomedical fields. Biomedical projects are increasingly adopting secondary data, and researchers are sharing more data through repositories, as journals and funding agencies often require researchers to share data in various formats [34]. In response to rapidly increasing needs, the emphasis of RDM services is inevitably on data creation, analysis, and sharing stages.

Although the data description, storage, and long-term preservation stages are less supported by the NIH Library, the significance of RDM will eventually require attention to these stages and suggests a potential need for the library's expansion of RDM services. Indeed, to fill this gap, the library plans to offer a new course, Data Management and Sharing, in Fall 2020. It is worth noting that, in addition to library services, the crosstab should include RDM services provided by other units in the institution, such as the IT department. Because not all RDM-related services can be provided solely by a library, it is useful to understand who supports RDM services and what areas can be supported by other units of an institution.

### Identify services that can be supported via partnerships

The gaps identified from the two models' crosstab may be due to the library's lack of resources. Key factors that have made some of the RDM services offered by the NIH Library successful are the library's flexibility, clear understanding of institutional context and users' specific needs, and institutional support from the NIH. Despite being comprised of only three librarians, the library's flexibility enables the data services team to easily collaborate with others who possess the necessary expertise to make RDM services available to users. Similarly, other libraries can build up project-based collaboration while maintaining existing functional structures if the staff includes skilled librarians rather than seeking expertise outside the library.

The library also draws on other units within the NIH and external partners with different skills that complement one another. Previous literature also highlights close collaboration between an institution's service units as key to the success of RDM services [11–14]. Such collaboration maximizes RDM service resources at the institutional level, reduces duplicated services, and publicizes services to a wider range of users. Using the NIH Library as a case shows that libraries do not have to stretch services beyond their capacity but instead can successfully work with other units that can supplement resources and knowledge. For example, the library expanded its educational services, particularly statistical applications training such as R, as a direct result of increased demand from users. On the other hand, CIT provides the institution's IT infrastructure; therefore, instead of providing similar services, it would be more important for the library to communicate with CIT to let them know the potential use of their services in data management and how CIT's services result in filling the crosstab.

Opportunities for collaboration also exist with external vendors or organizations. For example, the NIH Library works with software vendors for training on specific software purchased by the library and with the Carpentries for training in data skills and data literacy assessment. The NIH Library also brought in other NIH units and external partners to supplement their capacity (e.g., lack of human resources) and capabilities (e.g., data literacy assessment). Thus, rather than trying to serve all data management stages, it may be more efficient for a library to recognize their gaps in RDM services and identify ways to fill those gaps. As shown by the NIH Library, gaps can be reduced through collaboration at different levels if a library can identify partnerships for some types of RDM services.

### Get regular feedback from users

A number of existing studies share examples of current RDM services and researcher needs (e.g., [3–6], [10]). However, the types of services needed may differ by institution and research community. It is good to review best practices and service cases to improve a library's RDM services. However, to ensure successful reception of the services, improvements in and implementation of RDM services should be based on users' changing needs within the institutional environment and culture, as well as research community trends. For example, due to NIH researchers' busy schedules, the library provides multiple delivery options for its services, including in-person training, webinar training, one-on-one consultations, and training-the-trainee programs. These decisions regarding service formats may not apply to other institutions or libraries with different resources or user needs.

Depending on trends and priorities in research communities, the needs of researchers may change. Thus libraries should be flexible to changing needs and reflect their RDM services accordingly. The NIH Library was able to identify researchers' increasing needs for support of data sharing to respond to expectations from the research community. Also, the NIH Library actively seeks input from NIH researchers and feedback on their services. For instance, based on training participants' feedback, they prioritized their training sessions to include more R classes.

Examining the NIH Library as a case study enabled us to explore a health science library's RDM services based on OCLC's RDM services categories and the data lifecycle model, which reveals the landscape of a library's RDM services. Our findings provide a method for other health science libraries to determine which RDM services support users' RDM practices, identify gaps in services, explore how RDM services can be provided through partnership, and understand researchers' changing needs through RDM service assessment. This method also highlights the importance of having multiple assessment approaches to allow libraries to improve their services and realize the use and allocation of resources at an institutional level. However, a limitation of this study is its focus on only one specific case, which restricted the number of interviewees. Consideration of other research institutions for comparison may provide a more comprehensive understanding of library RDM services and future research models.

In conclusion, we examined institutional and library-provided RDM services according to OCLC's RDM services categories and the data lifecycle using the NIH Library as a case study. This study demonstrates the use of a crosstab analysis of these two models to categorize current RDM services and identify service gaps. The NIH Library provides an example for understanding RDM support within the institutional environment and potential expansion of RDM services. This study contributes to the existing literature in three ways. First, it analyzes RDM services through the lens of OCLC's RDM services categories and the data lifecycle to identify areas for new or improved services. Second, using the NIH Library as a case study, it demonstrates how to utilize a crosstab analysis of two models to analyze RDM services within an institution. Third, it highlights practical considerations for designing and implementing RDM services offered by health science libraries.

## Data Availability

Interview codes are available in the appendix. Full transcripts cannot be shared due to participant confidentiality concerns.

## SUPPLEMENTAL FILES

**Appendix 1** Semi-structure interview questions

**Appendix 2** Final codes used for analysis
