# Supplementary material for: Practical considerations for a library's research data management services: the case of the National Institutes of Health Library
Source: J Med Libr Assoc. 2021 Jul 1;109(3):450–8. doi: 10.5195/jmla.2021.995 (PMC8485941; doi:10.5195/jmla.2021.995)
Supplement: Supplementary file 1 — Appendix 1 Semi-structure interview questions [file jmla-109-3-450-s01.docx]

**Appendix 1.** Semi-structure interview questions

| **Question categories** | **Questions** |
| --- | --- |
| Personal information | Librarian's responsibility (job title)  Types of data services he/she provides |
| Library information | **Users**: User characteristics  **Services**: scope of RDM services (types and number of services), how the services are provided (scheduling), materials (e.g. workshop/training/tutorials etc.)  **Organizational Structure**: structure, collaboration experiences  **Infrastructure**: RDM infrastructure at the library and at NIH |
| RDM services in general | Who is responsible for data management support? Who do you communicate with mostly among various user groups?  What kinds of support do you provide in writing DMP? |
| Data creation | What kinds of support do you provide for data creation? Do you provide any tools and software for data creation? |
| Data description | What kinds of support do you provide for data description and documentation? Do you provide any tools and software for data description and documentation? |
| Data analysis | What kinds of support do you provide for data analysis? Do you provide any tools and software for data analysis? |
| Data storage | What kinds of support do you provide for data storage? Do you provide any tools and software for data description and documentation? |
| Data preservation | What kinds of support do you provide for long-term data preservation? |
| Publication and sharing | What kinds of support do you provide for data sharing?  What kinds of support do you provide to help users fulfill data sharing requirements for journals?  What kinds of support do you provide to help users fulfill data sharing requirements from NIH or other federal agencies? |
| Difficulties in data management and sharing | What are the issues that your users have in data management? What kinds of support do you provide for resolving those issues?  What are the problems your users have in data sharing? What kinds of support do you provide for resolving such problems? |
| Needs for library services | As a librarian, do you have any suggestions to improve current RDM services based on your experience with users?  What NIH Library's data services do users use?  Have you conducted user surveys or interviews to measure users’ satisfaction with education/training programs? |
