# Supplementary material for: Practical considerations for a library's research data management services: the case of the National Institutes of Health Library
Source: J Med Libr Assoc. 2021 Jul 1;109(3):450–8. doi: 10.5195/jmla.2021.995 (PMC8485941; doi:10.5195/jmla.2021.995)
Supplement: Supplementary file 2 — Appendix 2 Final codes used for analysis [file jmla-109-3-450-s02.docx]

**Appendix 2.** Final codes used for analysis

| **Main categories** | **Codes** |
| --- | --- |
| Library | Librarian’s responsibilities |
|  | Organizational structure |
| Users | User base |
|  | User characteristics |
|  | DMP |
| Institutional environment | Physical environment |
|  | As a federal agency |
| OCLC–Education services | Educational goal |
|  | Perception about online education |
|  | User feedback |
|  | Training classes |
|  | Common data elements |
| OCLC–Expertise services | Secondary data consultation |
|  | Data wrangling |
|  | Visualization |
|  | Writing services |
|  | Data management hw/sw |
|  | Difficulties |
| OCLC–Curation services | CIT services |
|  | Institutional DB |
|  | Public repositories |
|  | Data management hw/sw |
| Data lifecycle stages | Data creation |
|  | Data description |
|  | Data analysis |
|  | Data storage |
|  | Data preservation |
|  | Data sharing |
| Collaboration–within library | Flexibility |
| Collaboration–NIH units | Need for collaboration |
|  | Communication |
|  | Example cases |
| Collaboration–external partners | Need for collaboration |
|  | Example cases |
